# Publisher Correction: Combinatorial effects on gene expression at the *Lbx1/Fgf8* locus resolve split-hand/foot malformation type 3

**DOI:** 10.1038/s41467-023-38736-7

**Published:** 2023-05-18

**Authors:** Giulia Cova, Juliane Glaser, Robert Schöpflin, Cesar Augusto Prada-Medina, Salaheddine Ali, Martin Franke, Rita Falcone, Miriam Federer, Emanuela Ponzi, Romina Ficarella, Francesca Novara, Lars Wittler, Bernd Timmermann, Mattia Gentile, Orsetta Zuffardi, Malte Spielmann, Stefan Mundlos

**Affiliations:** 1 Max Planck Institute for Molecular Genetics, RG Development & Disease, Berlin, 14195 Germany; 2 Institute of Medical and Human Genetics, Charité Universitätsmedizin Berlin, Berlin, 10117 Germany; 3 Department of Computational Molecular Biology, Max Planck Institute for Molecular Genetics, Berlin, 14195 Germany; 4 Medical Genetics Unit, Department of Reproductive Medicine, ASL Bari, Bari, 70131 Italy; 5 Microgenomics Laboratory, Pavia, 27100 Italy; 6 Department of Developmental Genetics, Transgenic Unit, Max Planck Institute for Molecular Genetics, Berlin, 14195 Germany; 7 Sequencing Core Facility, Max Planck Institute for Molecular Genetics, Berlin, 14195 Germany; 8 Department of Molecular Medicine, University of Pavia, Pavia, 27100 Italy; 9 Institute of Human Genetics, Universitätsklinikum Schleswig Holstein Campus Kiel and Christian-Albrechts-Universität, Kiel, 24118 Germany; 10 Institute of Human Genetics, University of Lübeck, Lübeck, Germany; 11 Human Molecular Genomics Group, Max Planck Institute for Molecular Genetics, Berlin, 14195 Germany; 12 Berlin-Brandenburg Center for Regenerative Therapies (BCRT), Charité Universitätsmedizin Berlin, Berlin, 13353 Germany; 13 Present Address: Department of Pathology, New York University School of Medicine, Langone Health Medical Center, New York, NY 10016 USA; 14 Present Address: Kennedy Institute of Rheumatology, University of Oxford, Oxford, OX3 7FY UK; 15 Present Address: Centro Andaluz de Biología del Desarrollo, Consejo Superior de Investigaciones Científicas/Universidad Pablo de Olavide, Seville, 41013 Spain; 16 Present Address: Universität Innsbruck, Innsbruck, 6020 Austria

**Keywords:** Gene regulation, Chromatin structure, Limb development

Correction to: *Nature Communications* 10.1038/s41467-023-37057-z, published online 17 March 2023

In this Article, gene name labels for *Fbxw4*, *Poll* and *Dpcd* were misplaced in Figures 2f and 3c so as not to align with the correct element of the figure. These errors have been corrected in the HTML and PDF versions of the article.

