# Supplementary material for: Combinatorial effects on gene expression at the Lbx1/Fgf8 locus resolve split-hand/foot malformation type 3
Source: Nat Commun. 2023 Mar 17;14:1475. doi: 10.1038/s41467-023-37057-z (PMC10020157; doi:10.1038/s41467-023-37057-z)
Supplement: Supplementary file 1 — Supplementary information [file 41467_2023_37057_MOESM1_ESM.pdf]

SUPPLEMENTARY FIGURES

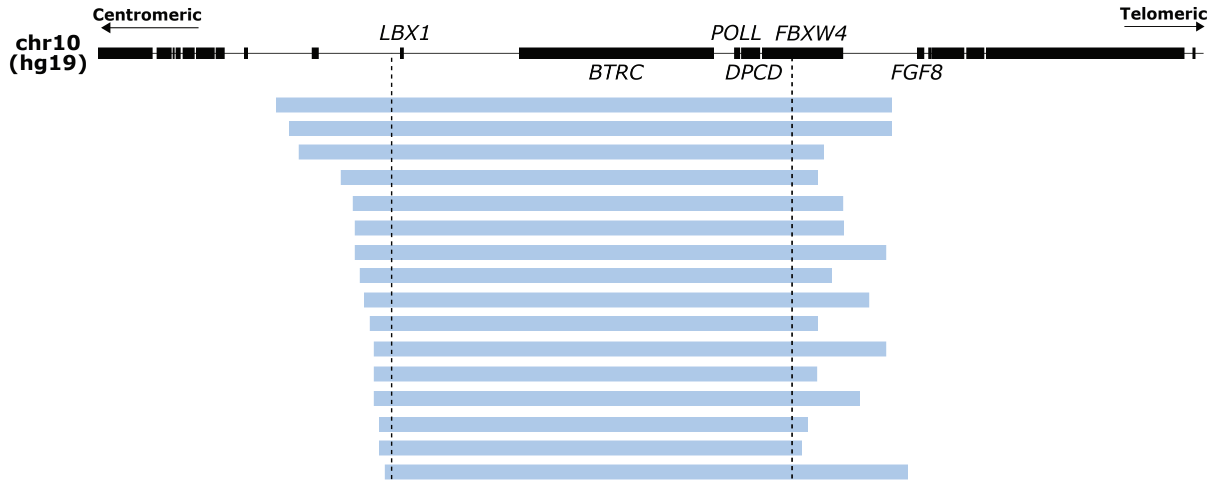

Supplementary Fig. 1 | Overview of in house SHFM3 duplications. All the identified duplications included *LBX1* at the centromeric side and excluded *FGF8* at the telomeric side. Dashed black lines indicate the minimal critical region of the duplications.

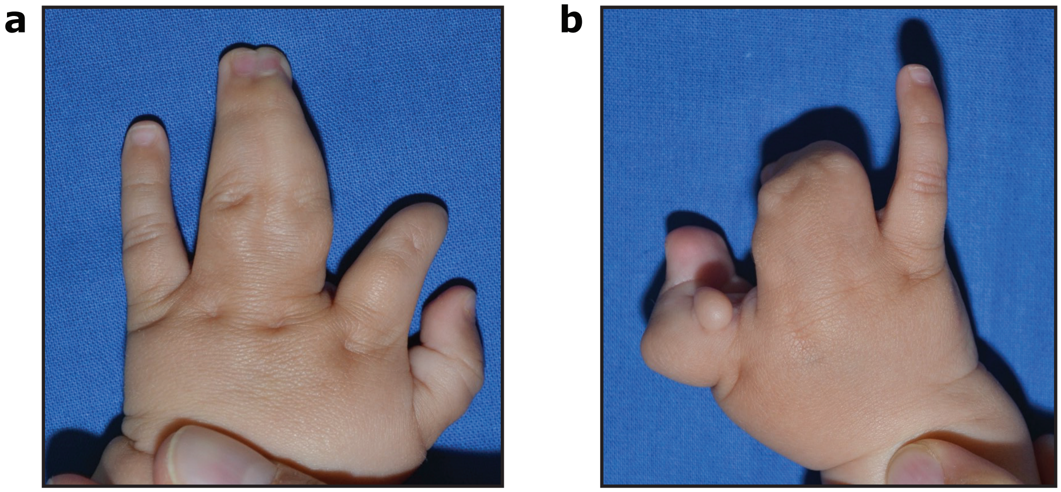

Supplementary Fig. 2 | Patient carrying an inversion at the *LBX1*/*FGF8* locus exhibited two phenotypes of the SHFM spectrum. a Left hand showing fusion of digit III and IV. **b** Right hand showing absence of central distal digits.

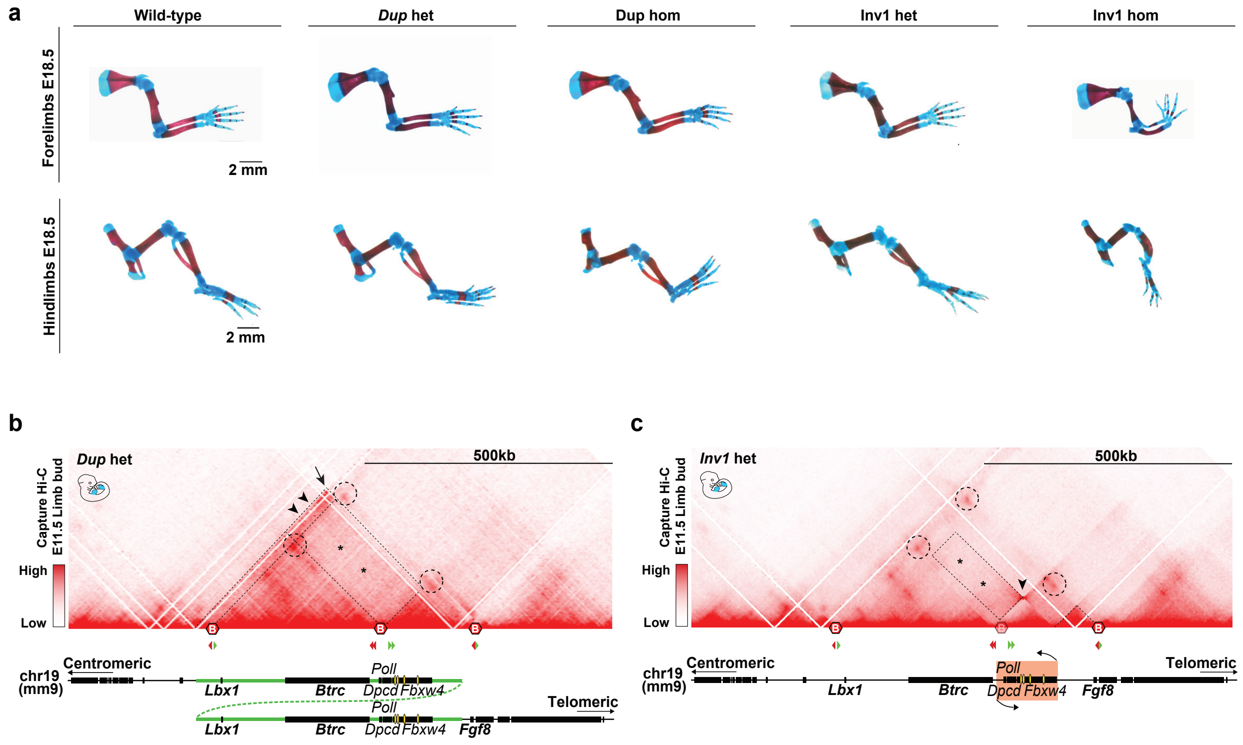

Supplementary Fig. 3 | Heterozygous *Dup* and *Inv1.* **a** Skeletal analysis of E18.5 forelimbs and hindlimbs stained with alcian blue (cartilage) and alizarin red (bone). Comparison between wild-type, heterozygous and homozygous highlighted the presence of underdeveloped limbs in homozygous *Inv1*. **b** cHi-C (n=1) of heterozygous *Dup* from E11.5 mouse limb buds. Positions of preserved wild-type interactions between boundaries are indicated by dashed circles, while the new contact reflecting the breakpoints of duplication is highlighted by a black arrow. Black arrowheads indicate a gain of contacts between the region containing the *Fgf8* AER enhancers and the region immediately flanking the centromeric side of the *Lbx1* TAD boundary. The rectangular dashed area highlighted by asterisks show increased interactions between the *Fgf8* AER enhancers region and the *Lbx1* TAD. **c** cHi-C (data are shown as merged signal of n=3 biological replicates) of heterozygous *Inv1* from E11.5 mouse limb buds. Dashed circles indicate the positions of the original wild-type interactions between boundaries, two of them (centromeric and telomeric) lost for the allele carrying the inversion upon reshuffling of the boundary between *Lbx1* and *Fgf8* TADs. The boundary involved in the inversion is shown as blurry. Dashed lines on the right point out the new smaller *Fgf8* TAD, now comprising only *Fgf8*. Black arrowhead highlights the bow tie configuration representative of the inverted regions. The rectangular dashed area highlighted by black asterisks show ectopic interactions between the *Fgf8* AER enhancers region and the *Lbx1* TAD.

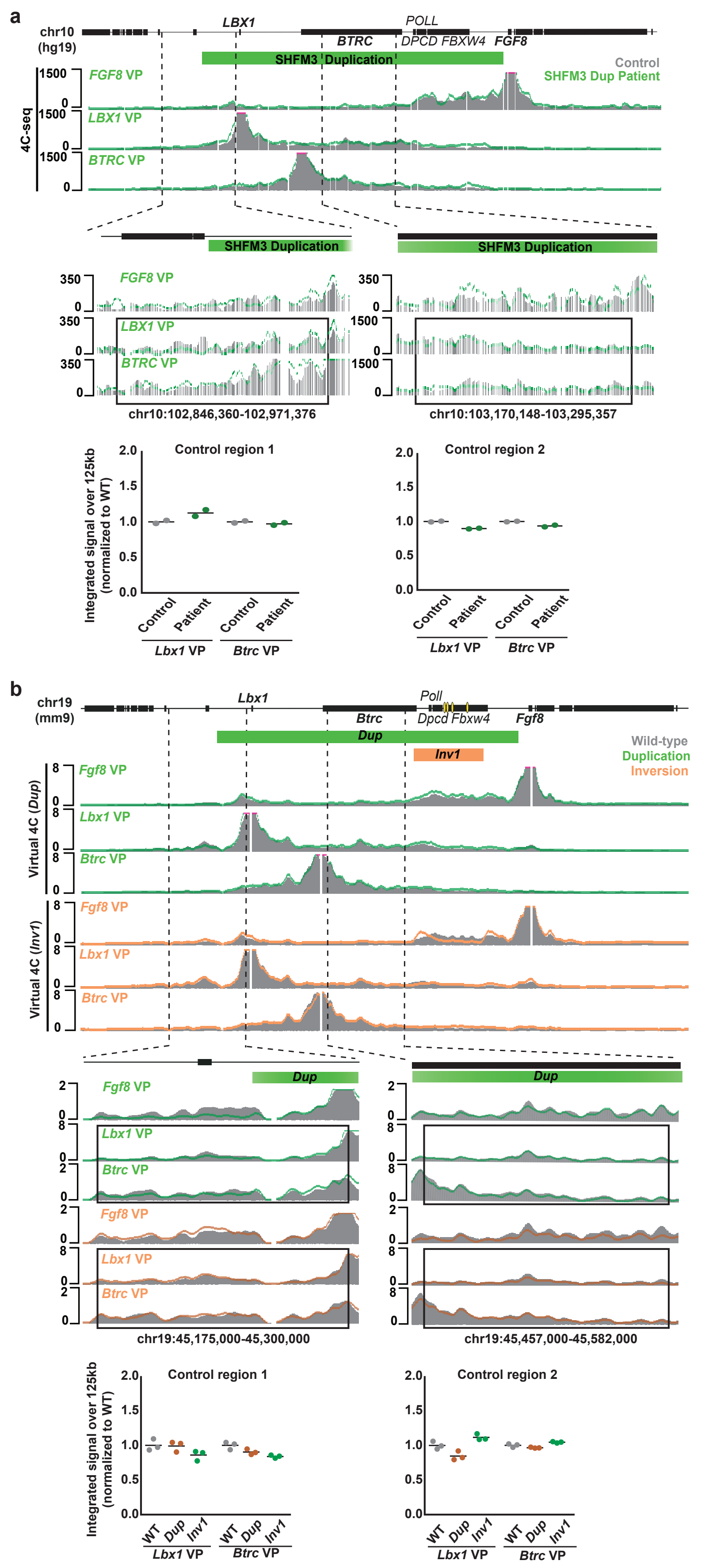
a
chr10
(hg19)
LBX1
POLL
BTRC
DPCD FBXW4
FGF8
SHFM3 Duplication
Control
SHFM3 Dup Patient
4C-seq
FGF8 VP
LBX1 VP
BTRC VP
1500
0
350
chr10:102,846,360-102,971,376
chr10:103,170,148-103,295,357
Control region 1
Control region 2
Integrated signal over 125kb
(normalized to WT)
2.0
1.5
1.0
0.5
0.0
Control
Patient
Lbx1 VP
Btrc VP
b
chr19
(mm9)
Lbx1
Poll
Btrc
Dpcd Fbxw4
Fgf8
Dup
Inv1
Wild-type
Duplication
Inversion
Virtual 4C (Dup)
Virtual 4C (Inv1)
Fgf8 VP
Lbx1 VP
Btrc VP
8
2
0
chr19:45,175,000-45,300,000
chr19:45,457,000-45,582,000
WT
Dup
Inv1

Supplementary Fig. 4 | Control regions from 4C-seq in SHFM patient fibroblasts and virtual 4C in mouse show no ectopic interactions. a,b 4C-seq data of human fibroblasts (**a**) and mouse virtual 4C (**b**) as in Fig. 3 but focusing on two control regions. Integrated signals and zoom-in view of two control regions of 125kb show no or very mild difference of signal. This confirms that the increased seen in Fig. 3 is specific to the region of interest and not due to a bias of 4C signal. Healthy human control and mouse wild-type (WT) samples are shown in grey, SHFM patient and mouse mutant carrying a duplication are shown in green, while the mouse mutant carrying an inversion is shown in orange.

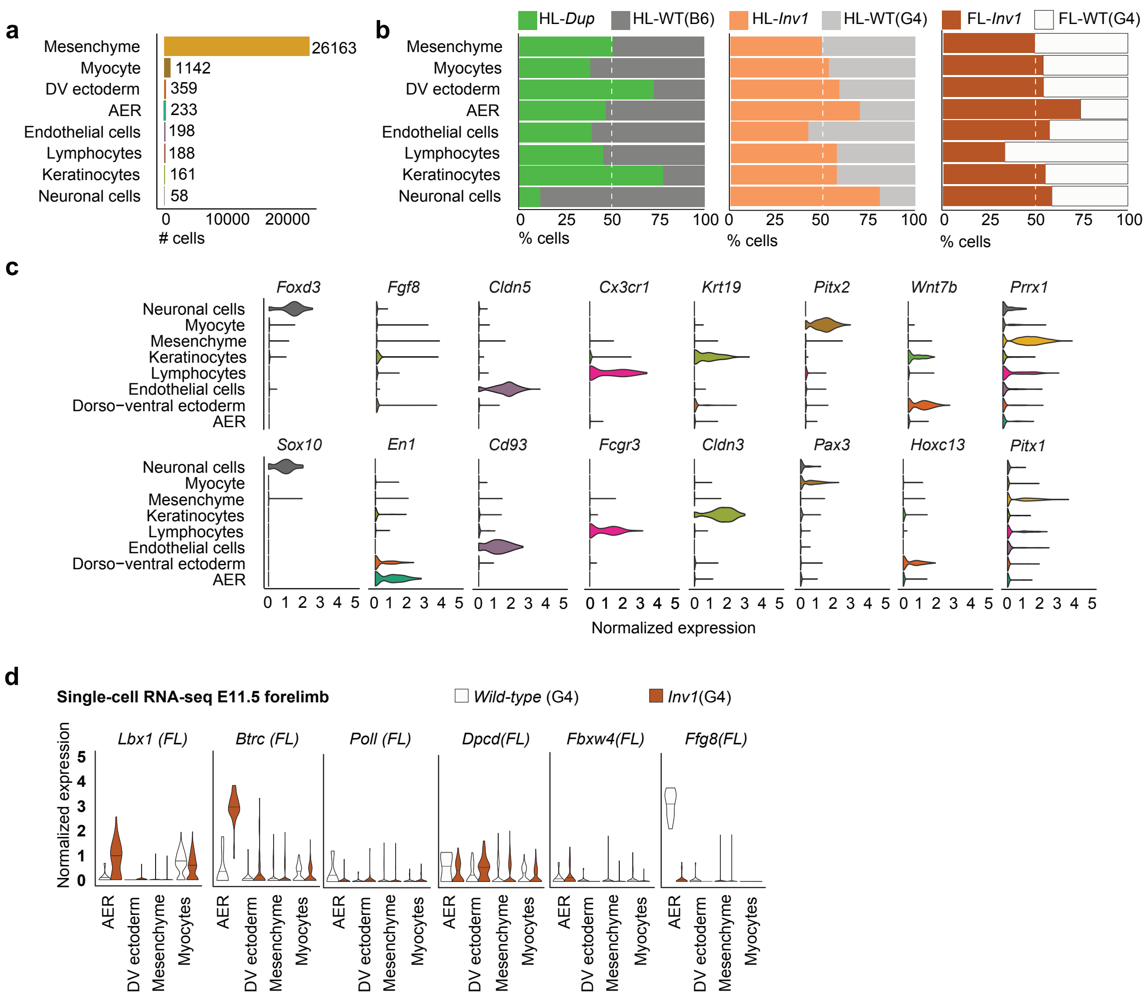

Supplementary Fig. 5 | scRNA-seq specifications. **a** Bar plot representing the number of cells per cluster as defined in Fig. 4B. These numbers correspond to the merged data of both wild-type, *Dup* and *Inv1* samples, from the scRNA-seq of E11.5 limbs. **b** Bar plot representing the % of cells per cluster as defined in Fig. 4B for each genotype separately. Some biases are observed, particularly for small cell population, as expected from scRNA-seq data. **c** Violin plots representing the expression of two marker genes per cluster that were used to confirm the clustering analysis shown in Fig. 4B. By example, AER cells are defined by high expression of *Fgf8* and *En1*. D Violin plot as in Fig. 4C from G4 wild-type and Inv1 mutant E11.5 forelimbs. **d** Violin plot representing the normalized expression of the 6 genes at the locus in AER, dorso-ventral (DV) ectoderm, mesenchyme and myocytes from E11.5 forelimb as in Figure4c. The Inv1 mutant (orange) was generated in a G4 (129sv x C57Bl6) background and thus compared to a G4 wild-type sample (white).

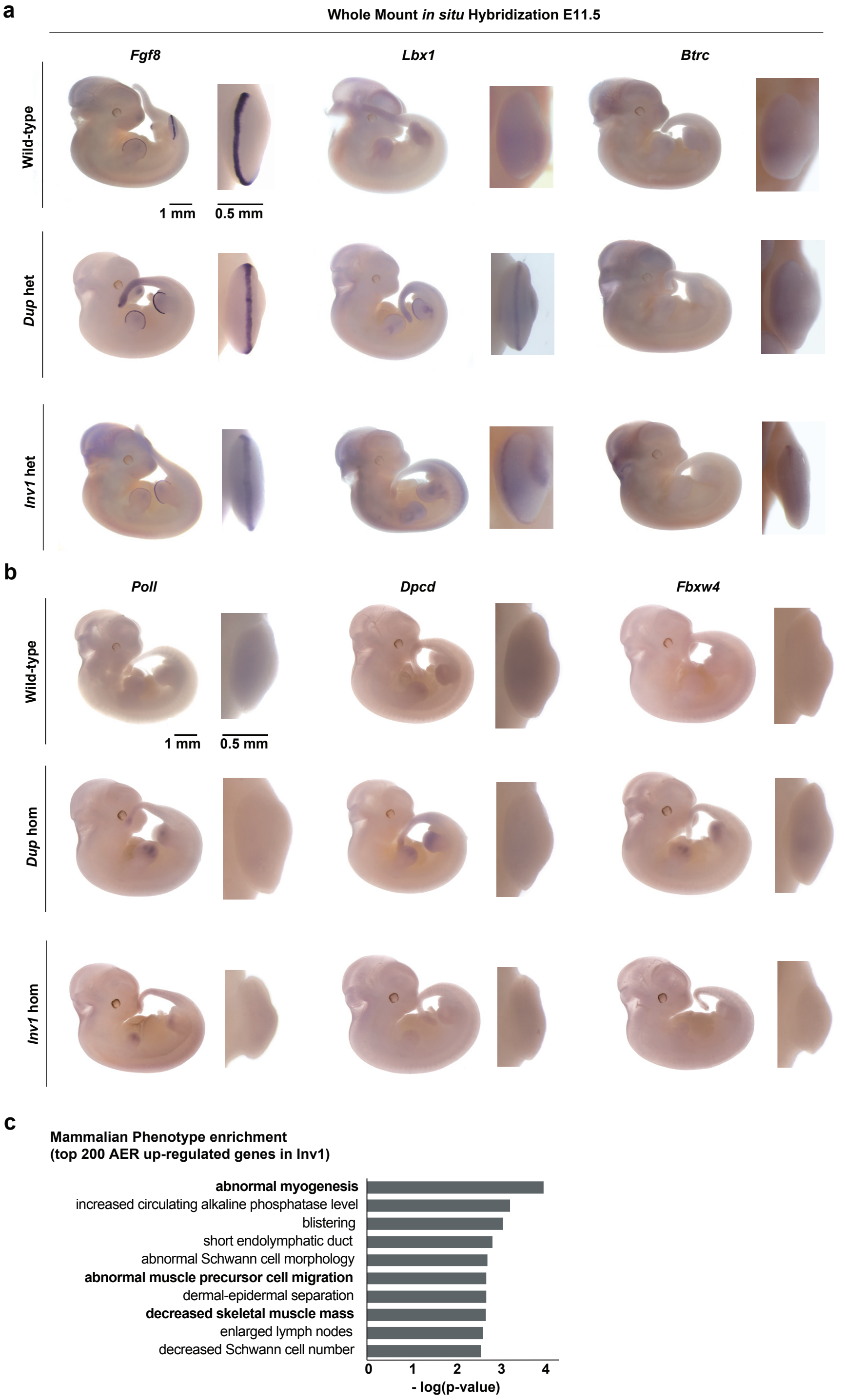

Supplementary Fig. 6 | No misexpression in the AER was detected for other genes at the *Lbx1-Fgf8* locus. a WISH for *Fgf8*, *Lbx1* and *Btrc* in heterozygous *Dup* and *Inv1*. Expression was checked and confirmed in at least 3 or more heterozygous embryos (at least *n*=3 biological replicates). **b** WISH for the other genes at the *Lbx1-Fgf8* locus for homozygous *Dup* and *Inv1*. Expression was checked and confirmed in at least 3 or more homozygous embryos (at least *n*=3 biological replicates). **c** GO analysis for the MGI Mammalian phenotype enrichment terms[59] using the top 200 genes that are up-regulated genes in the *Inv1* mutant compared to wild-type in the AER cells from the scRNA-seq data. The 10 most significant enriched terms are represented on a -log10 (p-value) scale. P-value was computed using a Fisher exact test.

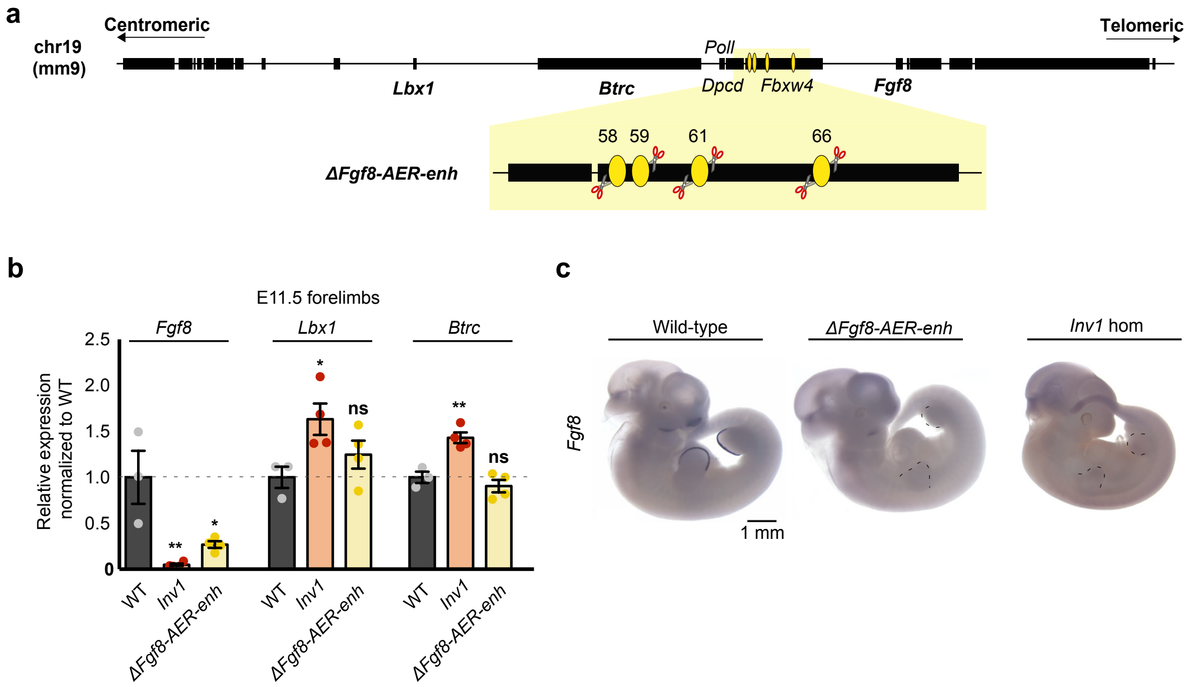

Supplementary Fig. 7 | Deletion of the 4 *Fgf8*-AER enhancers (58-59-61-66) leads to a strong loss of *Fgf8* expression in the E11.5 limb bud. a Scheme of the CRISPR-Cas9 mediated deletion of the 4 AER enhancers previously published (58-59-61-66)[28]. Enhancers are represented by yellow ovals. **b** RT-qPCR from E11.5 forelimbs showing the expression of *Fgf8*, *Lbx1* and *Btrc* in wild-type (n=3) and *Δ-Fgf8*-AER-enhancers (n=4) and *Inv1* homozygotes (n=4) mutants. *Fgf8* expression is affected in both mutants whereas *Lbx1* and *Btrc* expression are up-regulated only in the *Inv1* mutant as expected due to ectopic expression in the AER. Data are normalized to the WT and shown as means ± s.e.m. from *n*=3 or 4 replicates. Statistical analyses were performed by a one-way ANOVA with Bonferroni's multiple comparisons test (ANOVA p-value: *Fgf8* comparison, 0.0033; *Lbx1* comparison, 0.0598; *Btrc* comparison, 0.0007). Multiple comparison: * $p \leq 0.05$** $p \leq 0.01$. **c** WISH of wild-type and *Δ-Fgf8*-AER-enhancer and *Inv1* homozygotes E11.5 embryos showing *Fgf8* expression (n=3 embryos per genotype were analysed).

Supplementary Table 1: sgRNAs sequences

Dup _cen_sgF	caccgCCCAGGAATCGCCGATGACA
Dup _cen_sgR	aaacTGTCATCGGCGATTCCTGGGc
Dup _tel_sgF	caccgTTAAATTTCTCCAAGATAAG
Dup _tel_sgR	aaacCTTATCTTGGAGAAATTTAAc
Inv1 _cen_sgF	caccGGCCTCAATACACCTCCATG
Inv1 _cen_sgR	aaacCATGGAGGTGTATTGAGGCc
Inv1 _tel_sgF	caccGGTGACACCAGTATTCCTCG
Inv1 _tel_sgR	aaacCGAGGAATACTGGTGTCACCc
Fgf8AERenhKI _sgF	caccGCTTCCTGTATTGGACCCTG
Fgf8AERenhKI _sgR	aaacCAGGGTCCAATACAGGAAGCc
Inv2 _cen_sgF	caccgCTGACATTTAGCTGTGATCG
Inv2 _cen_sgR	aaacCGATCACAGCTAAATGTCAGc
Inv2 _tel_sgF	caccgTTAAATTTCTCCAAGATAAG
Inv2 _tel_sgR	aaacCTTATCTTGGAGAAATTTAAc
Inv1△Lbx1 _cen_sgF	caccgACCTCCGCAGTGTACCCATG
Inv1△Lbx1 _cen_sgR	aaacCATGGGTACACTGCGGAGGTc
Inv1△Lbx1 _tel_sgF	caccgCTCTCAGAAACTCGCAGCG
Inv1△Lbx1 _tel_sgR	aaacCGCTGCGAGTTTCTGAGAGc

Supplementary Table 2: primer sequences for PCR

Dup _cen_genoF	TGGTCATTGGAGCTCTGAGG
Dup _cen_genoR	GGATGTTTGGTCCAGTGTGC
Dup _tel_genoF	TTGATCAGGGGGTATGGTGT
Dup _tel_genoR	TCTGTTATTGGCGTGCAGTC
Inv1 _cen_genoF	CTCCCCACCCGTACTCTCTC
Inv1 _cen_genoR	GGGCCAGGCTAGGAGACTAA
Inv1 _tel_genoF	CAAAAACCACGTTGGTGGAC
Inv1 _tel_genoR	CTCGGCACCGACCTGTAA
Fgf8AERenhKI _outsideRHA+insert_genoF	CATTTCACTGCCTACCCATC
Fgf8AERenhKI _outsideRHA+insert_genoR	ACAGGGAGCTGGAGTCACTT
Fgf8AERenhKI _insideRHA+insert_genoF	CATTTCACTGCCTACCCATC
Fgf8AERenhKI _insideRHA+insert_genoR	GCAGGCAGACACGTGACAAAT
Fgf8AERenhKI _NOinsert_genoF	TTAGGTGAGCCCCAGAAGAAC
Fgf8AERenhKI _NOinsert_genoR	ACAGGGAGCTGGAGTCACTT
Inv2 _cen_genoF	ACTGCCCACATTTTGTAGCC
Inv2 _cen_genoR	CCTCCCAATCCTAGGGCTAC
Inv2 _tel_genoF	TTGATCAGGGGGTATGGTGT
Inv2 _tel_genoR	TCTGTTATTGGCGTGCAGTC
Inv1△Lbx1 _cen_genoF	AGACACTTGGGGTTCGCTTT
Inv1△Lbx1 _cen_genoR	GATTTCGGGGAAGTTTCAGG
Inv1△Lbx1 _tel_genoF	CATGTCTGATGACGGACGTCT
Inv1△Lbx1 _tel_genoR	ACTGTCGCGTTTTAAGGGAAA

Supplementary Table 3: primer sequences for qPCR copy number

Dup _5'flanking_F	AAGCTCCACTTGCCTTGGTA
Dup _5'flanking_R	TTCTCCAGCGGCATAGACTT
Dup _inside1_F	CTTTTCCCGGATCAATCTCA
Dup _inside1_R	GCCAGGGACATTTAGCAAGA
Dup _inside2_F	CACAAATCGTTTTCTTTGTCCA
Dup _inside2_R	TTAGCATCATTGAACGACATCC
Dup _inside3_F	TCTCCCCAAGACCATGAGAC
Dup _inside3_R	GTACTCGCTCCCACTTGAGG
Dup _inside4_F	CCAATCCTAGCCTCGATCAC
Dup _inside4_R	TCCTTATTCTGGCGGCTATG
Dup _inside5_F	CCTCATGTGCCCTTAAGGAA
Dup _inside5_R	CATGGAGCTAGGGACGAGAC
Dup _inside6_F	AACTGATTTAGAAGTGTCAGTCATGG
Dup _inside6_R	GCTGGTCCAGACCTTATCCA
Dup _inside7_F	AAAGTACGGGGAAAGGTGCT
Dup _inside7_R	TCCGGGTAGGAGTTTGATTG
Dup _inside8_F	TGGTTGCCGTCTGTCTGTAG
Dup _inside8_R	GGCGTCCAAACCTACCTGTA
Dup _inside9_F	AAAGAACCATGCACCTCCTG
Dup _inside9_R	CTCTCAGCTTCGAGGCACTC
Dup _inside10_F	GGTCACTGACATCCCCATCT
Dup _inside10_R	ACAGACGAGAGCCATCAAGC
Dup _inside11_F	GCCACCTCGAGAAAGATGAG
Dup _inside11_R	CTTCACCTTACCGGGAACAA
Dup _3'flanking_F	CTCCAGCACGATCTCTGTGA
Dup _3'flanking_R	AGGGAGGCTCCATAACACCT
Inv1 _5'flanking_F	AGAGCCCACGTAACCACAGT
Inv1 _5'flanking_R	CCTCCGTGAACTAACGCTTC
Inv1 _cen_wt1_F	GGACCACACTCACACTAATAGGC
Inv1 _cen_wt1_R	CACCCTGGCTACCTGTCAGT
Inv1 _cen_wt2_F	AGGCCTTAGAACCCTCAAAA
Inv1 _cen_wt2_R	CACCCTGGCTACCTGTCAGT
Inv1 _cen_mut_F	AGGCCTTAGAACCCTCAAAA
Inv1 _cen_mut_R	AGGAATGCTCTCCGGAAGTT
Inv1 _tel_mut_F	TCTTTCTGCCTAGGACATTGG
Inv1 _tel_mut_R	CGGAATACTGAAGCCCAGAC
Inv1 _3'flanking_F	GCCACCTCGAGAAAGATGAG
Inv1 _3'flanking_R	CTTCACCTTACCGGGAACAA
Inv2 _5'flanking1_F	TCATTTCGGAAGCTCTCAGG
Inv2 _5'flanking1_R	GGGACTTTTCATGCGTTTGT
Inv2 _5'flanking2_F	CACAAATCGTTTTCTTTGTCCA
Inv2 _5'flanking2_R	TTAGCATCATTGAACGACATCC
Inv2 _cen_wt1_F	GATGAAATATCTCCCGAGCAAC
Inv2 _cen_wt1_R	CGGCACAGTTGCTCATACAC
Inv2 _cen_mut_F	GATGAAATATCTCCCGAGCAAC
Inv2 _cen_mut_R	CAAGCCCACAGCTGTTATCA
Inv2 _tel_wt_F	CAAGCCCACAGCTGTTATCA
Inv2 _tel_wt_R	AAAGCCACAGACGGGTAATTT
Inv2 _tel_mut_F	CGGCACAGTTGCTCATACAC
Inv2 _tel_mut_R	AAAGCCACAGACGGGTAATTT

Inv2 _3'flanking1_F	CTCCAGCACGATCTCTGTGA
Inv2 _3'flanking1_R	AGGGAGGCTCCATAACACCT
Inv2 _3'flanking2_F	TCCAGCTCCAGACACTGAAC
Inv2 _3'flanking2_R	CGAAGCCTGTGACACTTGTT
Inv1△*Lbx1* _5'flanking1_F	TCGGGAGCAGATTTACGTTT
Inv1△*Lbx1* _5'flanking1_R	TTATGTGGCAATGAGCGAAG
Inv1△*Lbx1* _5'flanking2_F	TCCCGGGACACCTATAGTCC
Inv1△*Lbx1* _5'flanking2_R	AAAGCGAACCCCAAGTGTCT
Inv1△*Lbx1* _inside1_F	CTGTAATAGGCCGAGAGAGGTG
Inv1△*Lbx1* _inside1_R	GAGAGCGGCGAACACTACTAA
Inv1△*Lbx1* _inside2_F	GAAAGCGTTTCTCCAACTCG
Inv1△*Lbx1* _inside2_R	GATGGGATGACCATCTTTGG
Inv1△*Lbx1* _inside3_F	GCGACGAGAGAGTCAAGACC
Inv1△*Lbx1* _inside3_R	TGGAATCGAATTGGAGAAGAA
Inv1△*Lbx1* _inside4_F	TGGGAAGCTTGAGAAGGGTA
Inv1△*Lbx1* _inside4_R	TGGGATCATCAAAGTCCACA
Inv1△*Lbx1* _3'flanking1_F	CATTTGGATTCATGGCATTG
Inv1△*Lbx1* _3'flanking1_R	TGGCTTCTAGCATTCCCAAG
Inv1△*Lbx1* _3'flanking2_F	TCATTTCGGAAGCTCTCAGG
Inv1△*Lbx1* _3'flanking2_R	GGGACTTTTCATGCGTTTGT

Supplementary Table 4: RT-qPCR primer sequences

Fgf8 exon 4-5 (fron Marinic et al. 2013)	TATCGGTCTCCACAATGAGCTTCG
Fgf8 exon 4-5 (fron Marinic et al. 2013)	CCTGGCCAACAAGCGCATCAAC
Lbx1 exon 1-2 (fron Marinic et al. 2013)	GTGATTTTCGCCGTTTCTTG
Lbx1 exon 1-2 (fron Marinic et al. 2013)	AGACCTTTAAGGGGCTGGAG
Btrc exon 10-11 (fron Marinic et al. 2013)	ACAGAGACAGGCTGGTGGTGAGC
Btrc exon 10-11 (fron Marinic et al. 2013)	GCAGGCATGCTCCACACTCTATGTC
Rplp0 normalizer	TCCAGAGGCACCATTGAAATT
Rplp0 normalizer	TCGCTGGCTCCCACCTT
Rrm2 normalizer	CCGAGCTGGAAAGTAAAGCG
Rrm2 normalizer	ATGGGAAAGACAACGAAGCG

Supplementary Table 5: probes sequences for *in situ* hybridization

Lbx1 -ISH_fwd	Minina, E., Schneider, S., Rosowski, M., Lauster, R. & Vortkamp, A. Expression of Fgf and Tgfbeta signaling related genes during embryonic endochondral ossification. Gene Expr Patterns 6, 102–109 (2005).
Lbx1 -ISH_rev	
Btrc -ISH_fwd	CTCGTTAATGTTGCAGTATT
Btrc -ISH_rev	GTCTCCTCCCACGCAGTGGCT
Poll -ISH_fwd	CTTGGGCATGGGAAGGCTGA
Poll -ISH_rev	TGGCTGAGCTCCCTGAGAGC
Dpcd -ISH_fwd	GGCGGCTGAGTCAGAGTTACA
Dpcd -ISH_rev	CCTGAGTCTCTTGTATAAGG
Fbxw4 -ISH_fwd	GCCGAGCCGCGTGAAGCCGG
Fbxw4 -ISH_rev	AGCGGGATGAGGGCAGCGCAG
Fgf8 -ISH_fwd	Jagla, K. et al. Mouse *Lbx1* and human *LBX1* define a novel mammalian homeobox gene family related to the *Drosophila lady bird* genes. Mechanisms of Development 53, 345–356 (1995).
Fgf8 -ISH_rev	
