# Supplementary material for: Combinatorial effects on gene expression at the Lbx1/Fgf8 locus resolve split-hand/foot malformation type 3
Source: Nat Commun. 2023 Mar 17;14:1475. doi: 10.1038/s41467-023-37057-z (PMC10020157; doi:10.1038/s41467-023-37057-z)
Supplement: Supplementary file 3 — Description of Additional Supplementary Files [file 41467_2023_37057_MOESM3_ESM.pdf]

Description of Additional Supplementary Files

File Name: Supplementary Data 1
Description: DEseq analysis RNAseq

File Name: Supplementary Data 2
Description: 200 top DEG scRNAseq
