# Supplementary material for: Combinatorial effects on gene expression at the Lbx1/Fgf8 locus resolve split-hand/foot malformation type 3
Source: Nat Commun. 2023 Mar 17;14:1475. doi: 10.1038/s41467-023-37057-z (PMC10020157; doi:10.1038/s41467-023-37057-z)
Supplement: Supplementary file 6 — Reporting Summary [file 41467_2023_37057_MOESM6_ESM.pdf]

nature portfolio

Corresponding author(s): Stefan Mundlos, Giulia Cova

Last updated by author(s): Feb 28, 2023

Reporting Summary

Nature Portfolio wishes to improve the reproducibility of the work that we publish. This form provides structure for consistency and transparency in reporting. For further information on Nature Portfolio policies, see our Editorial Policies and the Editorial Policy Checklist.

Statistics

For all statistical analyses, confirm that the following items are present in the figure legend, table legend, main text, or Methods section.

n/a	Confirmed	
☐	☒	The exact sample size (*n*) for each experimental group/condition, given as a discrete number and unit of measurement
☐	☒	A statement on whether measurements were taken from distinct samples or whether the same sample was measured repeatedly
☐	☒	The statistical test(s) used AND whether they are one- or two-sided<br>*Only common tests should be described solely by name; describe more complex techniques in the Methods section.*
☐	☒	A description of all covariates tested
☒	☐	A description of any assumptions or corrections, such as tests of normality and adjustment for multiple comparisons
☐	☒	A full description of the statistical parameters including central tendency (e.g. means) or other basic estimates (e.g. regression coefficient) AND variation (e.g. standard deviation) or associated estimates of uncertainty (e.g. confidence intervals)
☐	☒	For null hypothesis testing, the test statistic (e.g. *F*, *t*, *r*) with confidence intervals, effect sizes, degrees of freedom and *P* value noted<br>*Give P values as exact values whenever suitable.*
☒	☐	For Bayesian analysis, information on the choice of priors and Markov chain Monte Carlo settings
☒	☐	For hierarchical and complex designs, identification of the appropriate level for tests and full reporting of outcomes
☒	☐	Estimates of effect sizes (e.g. Cohen's *d*, Pearson's *r*), indicating how they were calculated

Our web collection on statistics for biologists contains articles on many of the points above.

Software and code

Policy information about availability of computer code

Data collection	No commercial, open source or custom software/code was used to collect the data in this study.
Data analysis	- The size and position of the human structural variations (hg19/GRCh37) were converted to the mouse genome (mm9/NCBI37) using the UCSC liftOver (tool https://genome. ucsc.ed u/cgi-bi n/hgliftOver).<br>- sgRNAs were designed within close proximity of the duplication and inversion breakpoints, using the http://crispr.mit.edu/ platform to obtain candidate sgRNA sequences.<br>- RT-qPCR results were analyzed using the QuantStudio 7 Flex Real-Time PCR Software (version 1.7.1) (Applied Biosystems).<br>- The capture Hi-C SureSelect library probes were designed using the Sure Design on line tool from Agilent (https://earray.chem.agilent.com/suredesign/).<br>- The cHi-C data analysis, raw sequencing reads were processed with the HiCUP pipeline v0.8.1 (no size selection, Nofill: 1, Format: Sanger) using Bowtie2 v2.4.2 for mapping short reads to the reference genome mm9. Juicer tools v1.19.02 was used to generate binned and KR normalized contact maps from valid and deduplicated read pairs. For the generation of cHi-C maps, only read-pairs referring to the region of interest (chr19:44,440,001-46,400,000) and with MAPQ≥30 were considered. We used cHi-C maps with 5kb bin size. For the sample with a duplication, in order to consider the duplicated region explicitly, we created an additional version of the cHi-C map by applying LOIC normalization (python package iced v.0.5.1045) to raw count map, which has the aim to retain the effects of the increased copy number. For the LOIC normalization the copy number was set to 4 for all bins overlapping the duplication, and to 2 for the other bins. For five matrix rows/columns with low coverage, the count values were removed prior to LOIC-normalization.<br>- Data processing for the generation of the virtual 4C profile was performed with custom Java code using htsjdk v.2.12.0 (https://samtools.github.io/htsjdk/).

- For 4C-seq reads were mapped, normalized and smoothed with pipe4C using the reference genome GRCh37 and default settings. All viewpoints were performed in replicates and as quality measure >70% of reads were mapped within a size range of 1Mb and >80% within 100kb around the viewpoint.
- For RNA-seq reads were mapped to the mouse reference genome (mm9) using the STAR mapper (splice junctions based on RefSeq; options: –alignIntronMin 20–alignIntronMax 500000– outFilterMultimapNmax 5–outFilterMismatchNmax 10–outFilterMismatchNoverLmax 0.1).RNA-seq differential expression analysis was performed with the DEseq2 package.
- The Cell Ranger pipeline version 3 (10x Genomics Inc.) was used for each scRNA-seq sample to de-multiplex the raw base call files, generate the fastq files, and perform the alignment against a custom mouse reference genome mm9, to create the UMI count matrix. We used Scrublet to identify potential duplets in our dataset. Scurblet is available as online tool and has been incorporated into SPRING (https://klei ntools. h ms.harva rd.edu/tools/spring.htm l). Each sample dataset was normalized independently using the SCT method implemented in Seurat3 R package and then integrated the datasets using the Seurat3 Integrate Data function and considering the top 2000 most variable genes. To delimitate the major limb bud cell types, we used the Louvain algorithm implemented in the Seurat3 function FindClusters. For the AER cluster, we used the marker genes Fgf8 and En1. Differential gene expression was estimated by modelling the gene expression as a function of the genotype across cells. We fitted a quasi-Poisson distribution to calculate the effect on the gene expression distribution of each gene using the monocle3 strategy51. We tested that such effect was not equal to 0. We use this condition association effect to rank the genes and identified the pathways associated with the top 200 genes using the Enrichr tool from the Maayan lab52.

For manuscripts utilizing custom algorithms or software that are central to the research but not yet described in published literature, software must be made available to editors and reviewers. We strongly encourage code deposition in a community repository (e.g. GitHub). See the Nature Portfolio guidelines for submitting code & software for further information.

Data

Policy information about availability of data

All manuscripts must include a data availability statement. This statement should provide the following information, where applicable:

- Accession codes, unique identifiers, or web links for publicly available datasets
- A description of any restrictions on data availability
- For clinical datasets or third party data, please ensure that the statement adheres to our policy

All datasets have been deposited in the Gene Expression Omnibus (GEO) database and are accessible under GSE197404.
Previously published data used in this study include ChIP-seq data for CTCF from E11.5 mouse limb buds (GSE137335 or published previously under GSE84795).

Human research participants

Policy information about studies involving human research participants and Sex and Gender in Research.

Reporting on sex and gender

Fibroblasts from one male patient carrying a heterozygous SHFM3 duplication were used in this study to perform 4C-seq (Fig. 3). Pictures of hands with SHFM congenital limb malformations from one male patient carrying the newly here described heterozygous SHFM3 inversion were used in this study (Supplementary Fig. 2). The in-house reported SHFM3 duplications (Supplementary Fig. 1) are from both male and female patients. Sex and gender were not part of the study design.

Population characteristics

Patients carrying the in-house reported SHFM3 heterozygous duplications exhibited the classical SHFM phenotype with variable defects of the central rays of the autopod often together with syndactyly and/or aplasia/hypoplasia of the phalanges, metacarpals and metatarsals.
The male patient carrying a heterozygous SHFM3 duplication, whose fibroblast have been used to perform 4C-seq, was diagnosed at the age of 34 and exhibited the classical SHFM phenotype.
The patient carrying the inversion was diagnosed at the age of 1 and had fusion of digit III and IV in the left hand and absence of the central distant digits in the right hand.

Recruitment

Congenital limb malformations were observed at clinical genetic centers in Germany and in Italy. Given the nature of their phenotype, patients were offered additional diagnostic investigations in accordance with regulations for studies on human subjects at the respective centers. The investigators were not blinded to allocation during experiments and outcome assessment.

Ethics oversight

The study design and conduct complied with all relevant regulations regarding the use of human participants and was conducted in accordance with the criteria set by the Declaration of Helsinki. Informed consent to participate to the study and to publish clinical data was obtained from all patients (or their legal guardian). This study was approved by the Charité Universitätsmedizin Berlin ethics committee.

Note that full information on the approval of the study protocol must also be provided in the manuscript.

Field-specific reporting

Please select the one below that is the best fit for your research. If you are not sure, read the appropriate sections before making your selection.

☒ Life sciences ☐ Behavioural & social sciences ☐ Ecological, evolutionary & environmental sciences

For a reference copy of the document with all sections, see nature.com/documents/nr-reporting-summary-flat.pdf

Life sciences study design

All studies must disclose on these points even when the disclosure is negative.

Sample size	WISH experiments were performed from at least 3 independent biological mouse embryos. RT-qPCR experiments were performed using 3 independent biological replicates. Skeletal preparations were performed using at least 3 independent biological mouse embryos or pups. Phenotypic evaluation was performed using at least 3 animals per experiment. Capture Hi-C experiments were performed using 3 biological replicates per experiment plus one technical, except for Inv1 het where no technical replicate was produced, and for Dup het where only one biological replicate was used. RNA-seq analyses were performed using 2 biological replicates. scRNA-seq experiments were performed from one biological replicate. The specific number of replicates for each experiment was based on previous publications and experiments carried out in our laboratory.
Data exclusions	Samples/animals were included/excluded according to the genotype. Genotyping was established and verified in appropriate control experiments.
Replication	All experiments, except for scRNA-seq, were replicated 2 or 3 times, each of them successfully. For scRNA-seq one replicate is currently allowed given the nature of the technique, where data are processed as single cell, but that can be also collectively analyzed.
Randomization	Several founder animals for each mouse line were used for establishing line stock with variable intercrosses between single founder and C57BL/6 wild-type animals. Selection of animals for analysis and breeding was random. For all animal experiments, no choice of sample size was applied.
Blinding	Investigators were not blinded during experiments, the data collection was performed according to the stage of each sample since mouse breeding and analysis required knowledge about the genotype at hand.

Reporting for specific materials, systems and methods

We require information from authors about some types of materials, experimental systems and methods used in many studies. Here, indicate whether each material, system or method listed is relevant to your study. If you are not sure if a list item applies to your research, read the appropriate section before selecting a response.

Materials & experimental systems

n/a	Involved in the study	
☐	☒	Antibodies
☐	☒	Eukaryotic cell lines
☒	☐	Palaeontology and archaeology
☐	☒	Animals and other organisms
☒	☐	Clinical data
☒	☐	Dual use research of concern

Methods

n/a	Involved in the study	
☒	☐	ChIP-seq
☒	☐	Flow cytometry
☒	☐	MRI-based neuroimaging

Antibodies

Antibodies used	Anti-Digoxigenin (Roche Cat. #11093274910 - Fab fragments from an anti-digoxigenin antibody from sheep, conjugated with alkaline phosphatase).
Validation	Roche:<br>nucleic acid probes can be labeled very efficiently with digoxigenin and be used as hybridization probes in various membrane blot applications. After stringency washes, the blots are subjected to immunological detection using a anti-digoxigenin antibody conjugated to alkaline phosphatase and a chemiluminescent or color substrate. Colorimetric detection of a DIG-labeled probe is usually performed with the two colorless substrates BCIP and NBT. These substrates form a redox system. BCIP is oxidized by alkaline phosphatase to indigo by release of a phosphate group. In parallel, NBT is reduced to diformazan. The reaction products form a water-insoluble dark blue to brownish precipitate, depending on the type of membrane.<br><br>In our work:<br>overnight incubation with Anti-Dig antibody conjugated to alkaline phosphatase (1:5,000) at 4°C was followed by 8×30min washing steps at room temperature with TBST 2 (TBST with 0.1% Tween 20 and 0.05% levamisole–tetramisole) and left overnight at 4°C. Embryos were stained after equilibration in AP buffer (0.02M NaCl, 0.05M MgCl2, 0.1% Tween 20, 0.1M Tris–HCl and 0.05% levamisole–tetramisole in H2O) 3×20min, followed by staining with BM Purple AP Substrate (Roche). The stained embryos were imaged using a Zeiss SteREO Discovery V12 microscope and Leica DFC420 digital camera.

Eukaryotic cell lines

Policy information about cell lines and Sex and Gender in Research

Cell line source(s)	We used mouse embryonic stem cells (mESCs) from 129/SvxC57BL/6J F1 hybrid (G4) backgrounds. These cells were obtained

Cell line source(s)	from Dr. Anders Nagy (George et al., 2007). CD1 and DR4 Puromycin-resistant feeder cell lines, produced from CD1 and DR4 transgenic embryos, were used to culture the G4 cells. Experiments were done as previously reported by our lab (Kraft et al. 2015).
Authentication	Genetically modified mESCs were used to produce embryos using tetraploid and diploid aggregation. Genotyping confirmed the presence of the desired mutation.<br>CD1 and DR4 feeder cell lines were directly produced from mouse embryos originating from DR4 and CD1 mice crosses, respectively.
Mycoplasma contamination	All the cell lines were tested and were negative for mycoplasma contamination.
Commonly misidentified lines<br>(See ICLAC register)	No commonly misidentified cell lines were used.

Animals and other research organisms

Policy information about studies involving animals; ARRIVE guidelines recommended for reporting animal research, and Sex and Gender in Research

Laboratory animals	Mice from CD1, C57BL/6J, or (129/SvxC57BL/6J) Fl hybrid backgrounds were used in our study. Males and females from embryonic days E11.5 and E18.5 were used in our experiments. Routine bedding, food, and water changes were performed. Mice were housed in a centrally controlled environment with a 12-h light/12-h dark cycle, temperature of 20-22.2 Celsius , and humidity of 30-50%. All animal experiments followed all relevant guidelines and regulations.
Wild animals	The study did not involve wild animals.
Reporting on sex	Sex was not part of the study design.
Field-collected samples	The study did not involve samples collected from the field.
Ethics oversight	All animal procedures were conducted as approved by the local authorities (LAGeSo Berlin) under license numbers G0243/18 and G0176/19.

Note that full information on the approval of the study protocol must also be provided in the manuscript.
